# Triazole Fungicide Residues and Their Inhibitory Effect on Some Trichothecenes Mycotoxin Excretion in Wheat Grains

**DOI:** 10.3390/molecules26061784

**Published:** 2021-03-22

**Authors:** Tamer M. A. Thabit, Eman M. Abdelkareem, Nahla A. Bouqellah, Shokr A. Shokr

**Affiliations:** 1 Central Agricultural Pesticides Laboratory (CAPL), Agricultural Research Center (ARC), Giza 12611, Egypt; Tamerthabit144@gmail.com (T.M.A.T.); Dedetote1441@gmail.com (S.A.S.); 2 Saudi Arabia Grains Organization (SAGO), Riyadh 11471, Saudi Arabia; 3 Plant Pathology Research Institute, Agricultural Research Center (ARC), Giza 12619, Egypt; 4 Biology Department, Collage of Science, Taibah University, Al-Madinah Al-Munawarah 344, Saudi Arabia; Nahlataibah00@gmail.com

**Keywords:** DON, T-2, wheat, triazoles, GC-MS/MS, ELISA, MRM

## Abstract

Wheat is one of the global strategic crops and ranks third in terms of cereals production. Wheat crops are exposed to many fungal infections during their cultivation stages, some of which have the ability to secrete a number of toxic secondary metabolites that threaten the quality of the grains, consumer health, producer economics, and global trade exchange. Fifty-four random samples were collected from wheat which originated from different countries. The samples included 14 types of soft wheat to study the extent of their contamination with deoxynivalenol (DON) and T-2 toxin by auto-ELISA technology and r-biopharm microtiter plate. All samples were contaminated with DON toxin except one sample, and the values ranged between 40.7 and 1018.8 µg/kg^−1^. The highest contamination rates were in Lithuanian wheat and the lowest was in Indian wheat. Meanwhile, the highest average level of T-2 toxin contamination was in Lithuanian wheat grains with 377.4 µg/kg^−1^, and the lowest average was 115.3 µg/kg^−1^ in Polish wheat. GC-MS/MS and multiple reaction monitoring mode (MRM) were used to detect 15 triazole derivatives in the collected samples, which may be used to combat fungal diseases on wheat during the growing season. Only 9 derivatives were found: simeconazole, penconazole, hexaconazole, cyproconazole, diniconazole, tebuconazole, metconazole, fenbuconazole, and difenoconazole. These derivatives varied according to the origin of the wheat samples as well as their concentration, whereas another 6 derivatives were not detected in any samples. A direct inverse relationship was found between the DON concentration in the samples and the residues of simeconazole, penconazole, diniconazole, tebuconazole, metconazole, fenbuconazole, and difenoconazole, and the T-2 toxin showed the same relationship except for tebuconazole. The safe and rational use of some triazole derivatives may be a new approach and a promising strategy to not only reduce plant diseases and their problems, but also to get rid of some mycotoxins as grain contaminants.

## 1. Introduction

Wheat is one of the strategic crops of the world for feeding both humans and animals. Edible wheat is classified as common wheat (*Triticum aestivum*), club wheat (*T. compactum*), durum wheat (*T. durum*), and many other species [1]. Wheat is the third most important cereal crop, after maize and rice, in the food security basket, and the global wheat production reached to 762.2 metric tons in 2019/2020 [2,3].

Wheat plants are mostly attacked by several phytopathogenic fungi during cultivation, harvesting, and storage, which is associated closely with a reduction in production and quality. It may extend to consumer’s health as well as the economic value of the crop [4,5]. *Fusarium* pathogens are the most important seedborne fungi that have been correlated with wheat seedling blight and root rot. However, different species of *Fusarium* were found in both husks and grains at a ratio of 3:1, respectively [6,7]. *Fusarium graminearum*, *F. avenaceum*, *F. culmorum*, and *F. poae* were associated with wheat kernels and other cereal grains, meanwhile, *F. cerealis*, *F. equiseti*, *F. sporotrichoides*, and *F. tricinctum* were less frequent [8]. Most species of *Fusarium* fungi have the ability to secret various types of mycotoxins, such as fumonisins, zearalenone, and trichothecenes [9]. Trichothecenes have over 150 toxins produced by various fungi and are considered to incur general cytotoxic effects, having the ability to inhibit protein synthesis in ribosomes during all three stages of protein synthesis [10].

There are multiple protocols for fungal disease management, but fungicides are still at the forefront of these treatments and have the ability to control pathogenic fungi and their undesirable effects. Fungicide groups differ in active ingredients and most of them reduce growth and prevent sporulation as triazoles. The mode of action of triazoles depends on preventing the production of sterols, which are considered the main components of fungal cell membranes [11,12]. The triazoles group is one of the largest groups of fungicides, and the Bayer company produced the first of the triazoles in 1973. The newer triazoles are intrinsically more active than the previous ones and their effectiveness is related back to their original LD50 values. The triazole moiety group are some of the most important systemic fungicides that include a variety of compounds and contain 1,2,4-triazole moiety, which controls a wide range of fungal diseases on a wide range of crops, especially grains [13]. The study aims to investigate the level of deoxynivalenol and T-2 toxins that contaminate imported wheat grains, along with the remaining residue of various derivatives of triazole fungicides, which are widely used all over the world to reduce fungal diseases that attack wheat crops during the growing season, and also the possibility of generally predicting or indicating the form of the correlational relationship between them, which may be an effective direction for reducing mycotoxins that affect the quality of agricultural products, especially grains, during post-harvest and storage periods.

## 2. Results

Fifty-four samples of wheat of different origins were analyzed for triazole residues, which are considered one of the most important groups of fungicides used on wheat to control fungal infection. The validation method was performed at three fortification levels of 0.50, 0.05, and 0.005 mg/kg, with three replicates for each, recovery values ranged from 85–110, 86–108, and 71–96%, respectively, as shown in Table 1, and the average of the RSD% ranged from 5–17%. Values of the limit of detection (LOD) and limit of quantitation (LOQ) were statistically calculated and ranged from 0.002–0.004 and 0.006–0.012 ppm, respectively. MRL (maximum residue level) varied from one compound to another. The compounds that had no MRL yet performed, such as simeconazole, hexaconazole, azaconazole, diniconazole and etaconazole had the 0.01 ppm level considered as the lowest determination and MRL level, while for penconazole and propiconazole, 0.05 ppm was considered the lowest determination and MRL level as reported by the European commission in Table 1.

All samples were analyzed in two replicates and the final results are shown in Table 2. The RSD% of the results ranged from 8–22%, and the average of the contaminated samples of tetraconazole, azaconazole, etaconazole, propiconazole, epoxiconazole, and bromuoconazole were below the limit of quantitation in all samples, while simeconazole, penconazole, hexaconazole, cyproconazole, diniconazole, tebuconazole, metconazole, fenbuconazole, and difenoconazole were detected in 4, 27, 24, 10, 11, 24, 4, 7, and 4 samples of all the 54 samples analyzed, respectively. Most of the analyzed samples did not exceed the MRL individually, but their average may exceed it. Samples have been collected from different shipments during the season and vary from one shipment to another according to area variation and their distribution in the country of origin. The results exceeded the MRL for hexaconazole and diniconazole in samples of all origins, but when comparing them with the Japanese MRL levels database by the Japan Food Chemical Research Foundation, the results were 0.1 ppm for hexaconazole, and so fall within the safe limit.

The results of the Table 3 indicate that DON was found in the most of the tested wheat samples (53 samples) for both soft wheat samples collected from different origins such as the USA, Germany, France, India; and hard wheat samples collected from Canada, Germany, Australia, Lithuania, Poland, and Brazil. The highest concentration of DON was found in Lithuanian wheat samples, with an average value of 1018.8 µg/kg^−1^, followed by Canadian wheat with an average of 870.5 µg/kg^−1^. Whereas soft Indian wheat samples recorded the lowest DON contamination rate, with an average of 40.7 µg/kg^−1^ per 3 samples. T-2 toxin was also found in varying proportions in all the 54 wheat samples tested. The highest level of contamination was found in Lithuanian hard wheat, with an average of 377.4 µg/kg^−1^ for four samples, followed by French soft wheat with an average of 132.4 µg/kg^−1^ for six samples. The hard Polish wheat samples (13 samples) recorded the lowest levels of contamination with T-2 toxin at 115.3 µg/kg^−1^.

**Figure 1:**
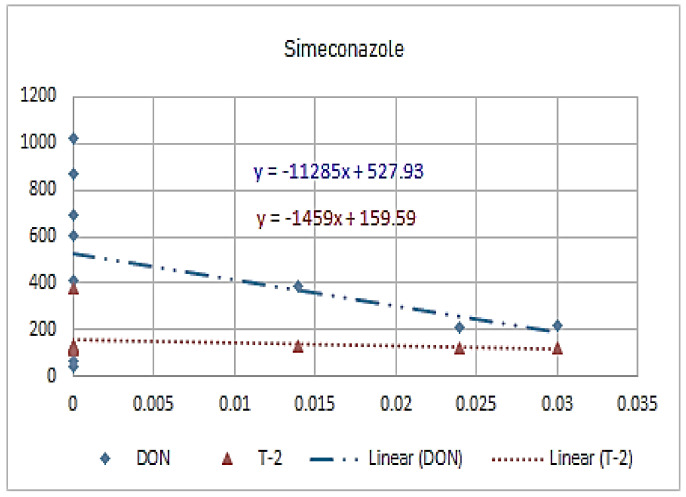
The relation between simeconzole residue (ppb) and DON and T-2 toxin (ppb) in wheat samples.

**Figure 2:**
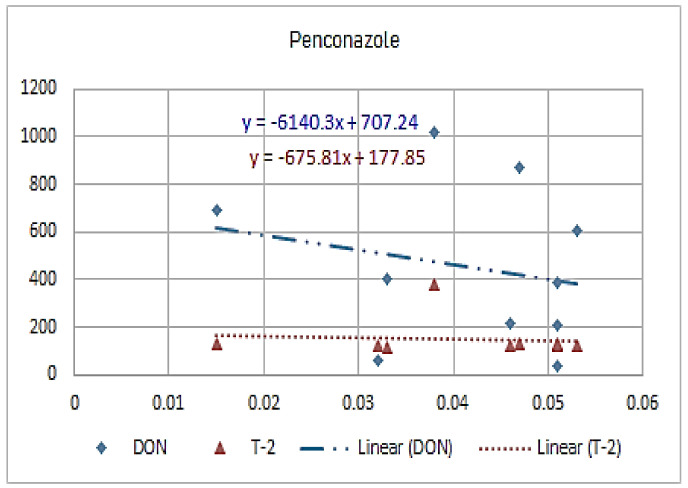
The relation between penconazole residue (ppb) and DON and T-2 toxin (ppb) in wheat samples.

**Figure 3:**
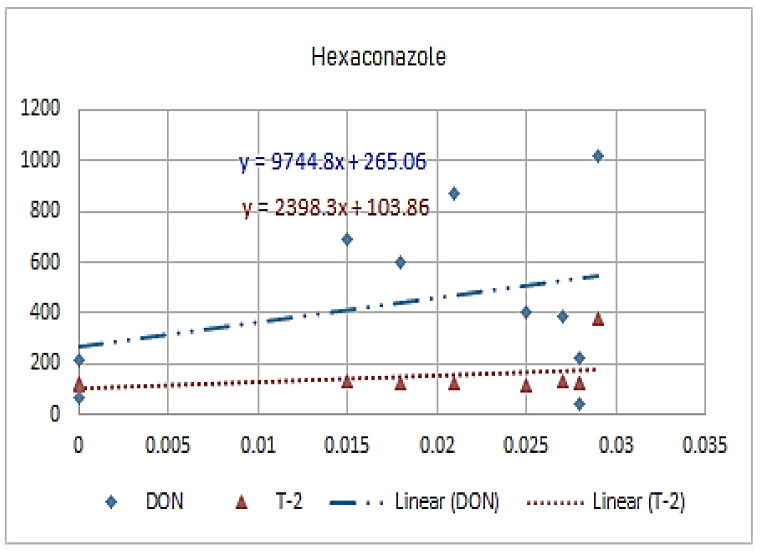
The relation between hexaconazole residue (ppb) and DON and T-2 toxin (ppb) in wheat samples.

**Figure 4:**
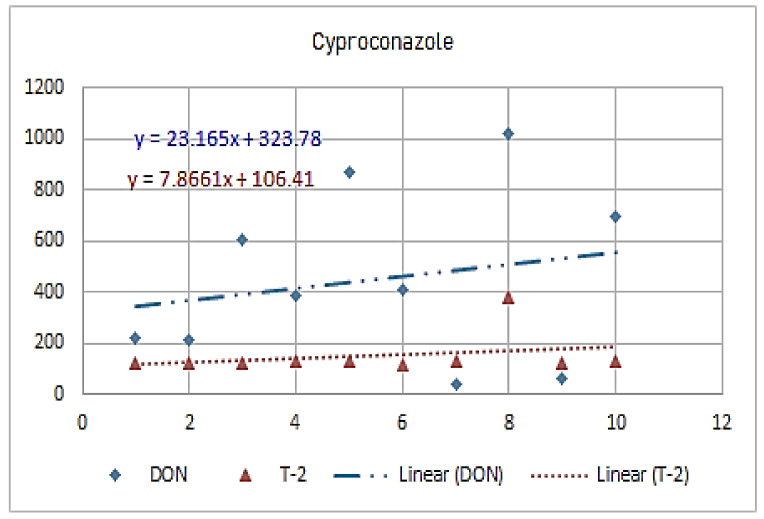
The relation between hyproconazole residue (ppb) and DON and T-2 toxin (ppb) in wheat samples.

**Figure 5:**
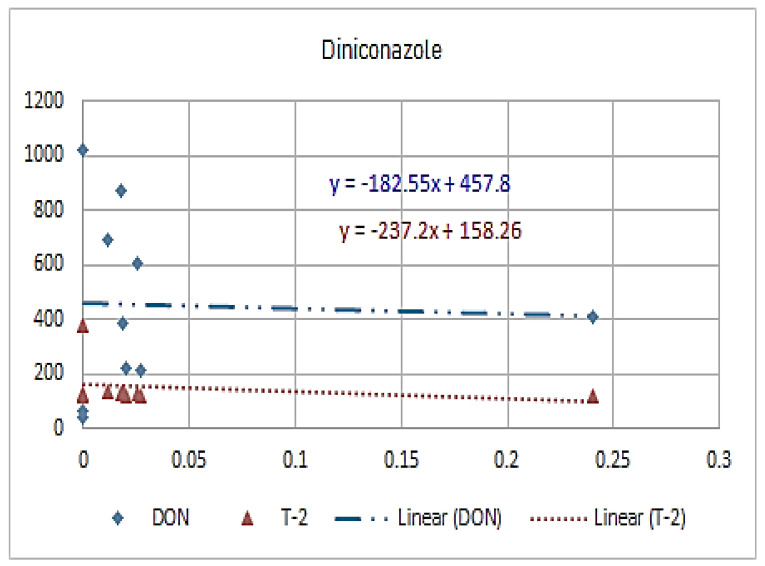
The relation between diniconazole residue (ppb) and DON and T-2 toxin (ppb) in wheat samples.

**Figure 6:**
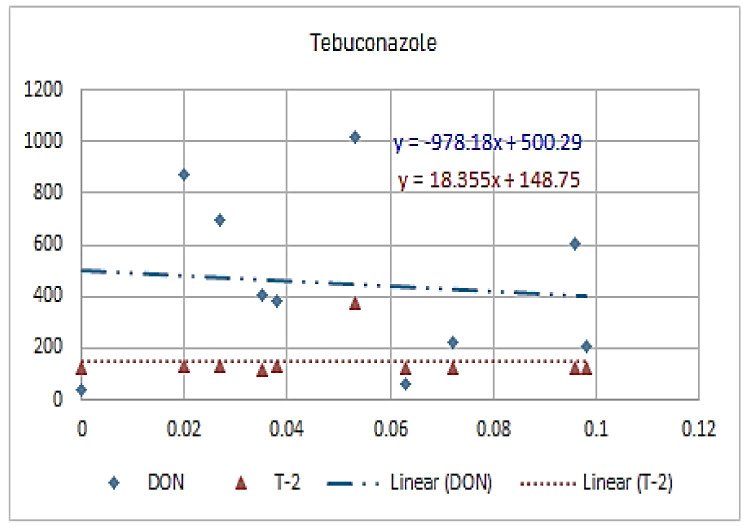
The relation between tebuconazole residue (ppb) and DON and T-2 toxin (ppb) in wheat samples.

**Figure 7:**
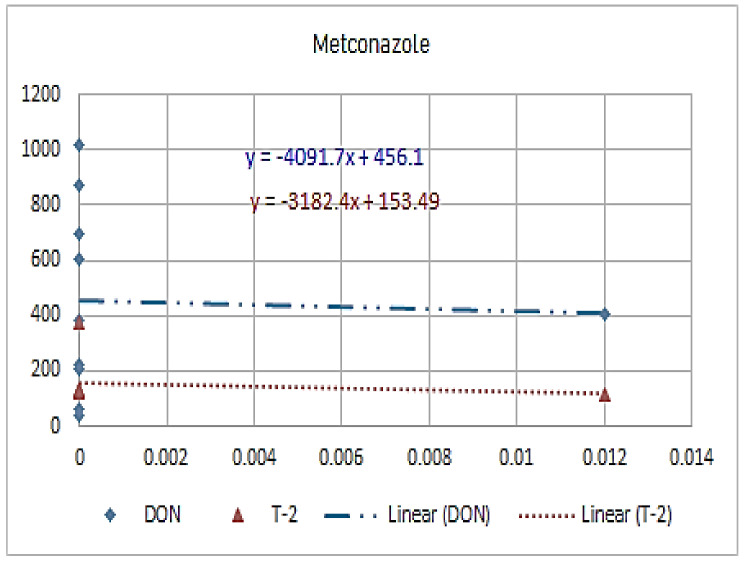
The relation between metaconazole residue (ppb) and DON and T-2 toxin (ppb) in wheat samples.

**Figure 8:**
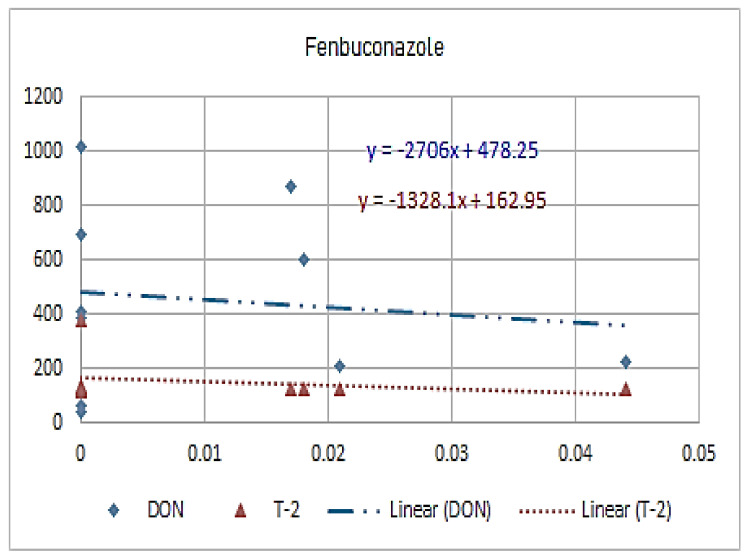
The relation between fenbuconazole residue (ppb) and DON and T-2 toxin (ppb) in wheat samples.

**Figure 9:**
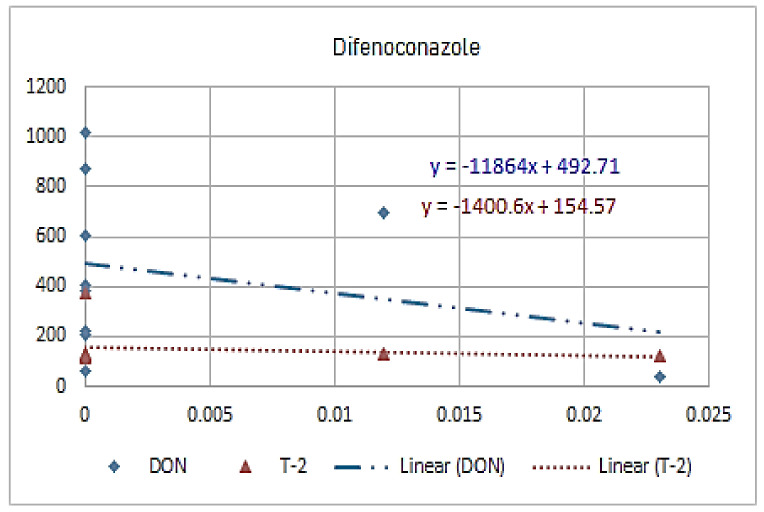
The relation between difenoconazole residue (ppb) and DON and T-2 toxin (ppb) in wheat samples.

Figure 1, Figure 2, Figure 3, Figure 4, Figure 5, Figure 6, Figure 7, Figure 8 and Figure 9 indicate that there is an inverse relationship between the concentration of different triazole derivative residues (simeconazole, penconazole, diniconazole, tebuconazole, metconazole, fenbuconazole, difenoconazole) in the wheat tested and DON toxin contamination with a different correlation value, while there was no correlation with both hexaconazole and cyproconazole. The same inverse correlation was noticeable between T-2 toxin and triazole derivative residues (simeconazole, penconazole, diniconazole, metconazole, fenbuconazole, and difenoconazole), but no such relationship existed with hexaconazole, cyproconazole, and tebuconazole.

## 3. Discussion

Wheat crops are exposed to attack by several fungal pathogens, especially on the shoots and spikes, causing blight, spots, streaking, rust, and smuts. Triazole compounds are considered one of the many available and recommended fungicides for controlling diseases in most wheat-growing areas and are used on a large scale to combat fungal pathogens such as *Fusarium* head blight, *Septoria tritici* blotch, leaf rust, and wheat blast [14,15].

Fifty-four samples of imported wheat grains were collected in Saudi Arabia, of which 14 samples of soft wheat and 40 samples of hard wheat were used for qualitatively and quantitatively estimating the derivatives of triazole fungicide. The results showed that neither tetraconazole, azaconazole, etaconazole, propiconazole, epoxiconazole, nor bromuconazole were detected in all the tested samples. Meanwhile, there were residues of nine of the triazole derivatives, namely simeconazole, penconazole, hexaconazole, cyproconazole, diniconazole, tebuconazole, metaconazole, fenbuconazole, and difenoconazole. The detected triazole derivatives varied in retention time (RT), ion transitions, and collision cell energy (C.E), as well as the % recovery at the levels of 0.5, 0.05, and 0.005. Meanwhile, the maximum residue level (MRL) ranged between 0.01 and 0.6, according to allowable European limits [16].

The wheat samples of different origins were all contaminated with DON toxin, except one sample of Brazilian origin. The contamination level of the tested samples with DON toxin varied and ranged between 40.7 and 1018.8 µg/kg^−1^. The contamination average of DON toxin per different origins was less than the regulation limits, which are estimated at 1250 µg/kg^−1^, according to the European Commission in 2006, but individually there were some of the samples in which the levels of DON toxin were more than the limit, such as the Canada, Lithuania and Brasilia samples [17]. The variation in DON toxin contamination may have been due to different factors such as environmental factors, agricultural treatments, and fungicide application during growing, which may have played a role in reducing fungal infection and doing what is known as mycotoxin decontamination [18,19,20]. Moreover, the wheat samples tested were all contaminated with T-2 toxin. The contamination level with T-2 toxin varied and ranged between 98.1 and 1107.0 µg/kg^−1^, whereas the permissible EU limits are from 400 to 1000 for cereals [20,21].

## 4. Materials and Methods

### 4.1. Samples Collection

Fifty-four samples, 2 kg each, of wheat imported by Saudi Arabia from countries of different origin from the harvest season of 2017/2018 were collected. Random representative samples were prepared according to the procedure described in the SANCO/12571/2013 document [22]. Samples were kept frozen upon arrival to the laboratory and the samples were then finely homogenized, soaked in liquid nitrogen, and ground prior to analysis process. The number of samples, the country of origin and their codes are noted in Table 4.

### 4.2. Recovery Experiments and Method Validation

Wheat samples free from pesticides were used in the validation studies and in the matrix-matched calibration standards preparation. The validation method was performed at three fortification levels: 0.50, 0.05, and 0.005 mg/kg, with three replicates for each, and the results are shown in Table 1. Working standard solutions at 1000 mg/L containing all the pesticides used for the validation method were prepared in acetonitrile. Six matrix-matched calibration standards at 3, 5, 10, 50, 100, and 250 ng/g were prepared with five replicates for each. Values of the limit of detection (LOD) and limit of quantitation (LOQ) for the analytical method used were estimated from the following equations as clarified in ICH (2005) [23].

LOD = 3.3 SD/b(1)

LOQ = 10 SD/b(2)

SD: the residual standard deviation of the regression line or standard deviation of y-intercepts of the regression line, b: The slope of regression line

### 4.3. Extraction and Cleaning-Up Method

Buffered QuEChERS procedure was used in the extraction and cleaning-up of fungicide residues [24]. As wheat is a dry matrix and a little bit high in fat, the steps mentioned by Mastovska et al., (2010) were used to raise the method’s sensitivity and output [25]. Five grams of finely ground wheat sample were placed in a 50 mL Teflon centrifuge tube, 10 mL of cold D.I. water was added, shaken carefully, and the mixture was allowed to swell and settle for 20 min. Ten mL of 1% HAc (glacial) in MeCN (*v*/*v*), was added and mixed well. TPP (triphenyl phosphate) as an internal standard (IS) at a rate of (200) ng/g sample was added and mixed well. Dispersive clean-up was done by adding six grams of activated anhydrous MgSO_4_ and 1.5 g of anhydrous NaOAc together and hand shaking vigorously as fast as possible for one min, it was then shaken with an orbital shaker for 60 min. Tubes were centrifuged at 5000 rpm for 10 min. Five mL of the upper layer were precisely transferred into a 15 mL centrifuge tube and kept in a deep-freezer for 30 min., then 750 mg of activated anhydrous MgSO_4_, 250 mg of PSA, and 250 mg of C18 were added to the cold extract and mixed well for one min. Tubes were centrifuged at 5000 rpm for 5 min. Four mL were transferred from the cleaned extract and evaporated with a Turbovap evaporator under N_2_ at 40 °C till the lowest volume (0.2–0.3 mL), then the volume was adjusted to one mL with toluene.

### 4.4. Triazoles Determination

A GC-MS/MS system of Agilent (model 7890B-7000C, Santa Clara, CA, USA) was operated in multiple reaction monitoring mode (MRM) with two ion transitions to obtain the maximum sensitivity for the detection of the target molecules, the mass transitions used are presented in Table 1. A HP-5MS capillary column (30 m × 250 µm × 0.25 µm) from J&W Scientific (Forson, CA, USA) was used for pesticide residue analysis, using the multi-mode inlet at 280 °C in splitless mode. The oven was programmed at 150 °C for two min, ramped at 3 °C/min^−1^ to 200 °C, then ramped to 280 °C at 8 °C/min^−1^ and then held for 10 min., the carrier gas was Helium (He) at a flow rate of 1 mL/min^−1^, with a retention time (Rt) as shown in Table 1. MS was operated in electron impact ionization mode (EI). The transfer line, MS source, quad1, and quad2 temperatures were 280, 300, 180, and 180 °C, respectively. Helium (He) quenching gas and N2 collision gas were used at 2.25 and 1.5 mL/min^−1^, respectively. The system was back flushed at 300 °C at 50 psi for five min., and the method retention time was locked to chlorpyrifos-methyl at 13.093 min. The Rt of the TTP used as IS was 24.162 min.

### 4.5. Trichothecenes Determination

Trichothecenes toxins were estimated according to Tima et al., (2016) [26]. 5 g of ground samples were individually weighted in a suitable container with 25 mL of 75% HPLC grade methanol. The sample was shaken vigorously for 3 min and then the extract was filtrated through a Whatman filter No.1. One mL of filtrate extract was diluted with 1 mL of distilled deionized water.

Auto-ELISA (ChemWell, Awareness Technology Inc., Palm City, FL, USA) and RIDASCREEN^®^Enzyme immunoassays software (r-biopharm, Pfengstadt, Germany) were used to conduct the procedures of DON toxin determination. 50 µL of standard (50, 100, 200, and 400 µL/L^−1^) and the prepared samples were injected into separate wells of a microtiter plate No. R5902 (r-biopharm, Germany) accredited from AOAC. Then, 50 µL of enzyme conjugate was added to the bottom of each well and 50 µL of anti-DON toxin antibody solution was added to each well and gently mixed by shaking the plate and incubating it for 10 min at 20 °C. After incubation, all the remaining liquid was removed from the wells, and they were refilled with 250 µL of distilled deionized water per well. The well was emptied again by removing all remaining liquid and this step was repeated two more times. Then, 100 µL of substrate/chromogen was added to each well, mixed gently, and incubated for 5 min at 20 °C. Finally, 100 µL of stop solution was added to each well, mixed gently by shaking, and the absorbance was measured at 450 nm. The measuring was done through 10 min of stop solution addition and the measuring range was 0.2–6.0 ppm. T-2 toxin estimation was similar to the DON protocol, with the kit being replaced by a No. R5302 (r-biopharm, Germany), which has a special microtiter plate, enzyme conjugate, antibody solution, and substrate/chromogen for T-2 toxin.

## Figures and Tables

**Table 1:** Separation and validation data of the studied fungicides.

Compound Name	Time Segment	Rt	Ion Transitions		C.E	Recovery %			RSD% Ave.	R^2^	MRL ppm	LOQ ppm	LOD ppm
						0.5	0.05	0.005					
Simeconazole	1	13.272	121	101.1	10	86.2	87.3	71.86	8–14	0.992	0.01 *	0.01	0.003
			121	75.1	25								
Tetraconazole	1	16.328	170.9	136	10	95.3	92.88	91.97	5–9	0.998	0.1	0.01	0.003
			336	217.9	20								
Penconazole	2	17.478	248	192.1	15	105.1	92.45	91.55	7–11	0.995	0.05 *	0.006	0.002
			248	157.1	25								
Hexaconazole	2	19.986	256	82.1	10	96.6	102.83	96.7	9–15	0.994	0.01 *	0.006	0.002
			231	175	10								
Azaconazole	2	21.012	217	173.1	15	85.2	86.13	78.88	9–12	0.988	0.01 *	0.01	0.003
			219	175	15								
Cyproconazole	3	21.369	139	111	15	98.2	98.89	94.37	10–15	0.995	0.1	0.01	0.003
			222	125.1	15								
Diniconazole	3	22.031	267.9	232.1	10	107.3	108.52	96.57	10–17	0.995	0.01 *	0.006	0.002
			269.9	232	10								
Etaconazole	3	22.102	173	145	15	110.1	107.2	96.11	11–16	0.998	0.01 *	0.01	0.003
			173	109	30								
Propiconazole	4	23.631	172.9	145	15	104.5	91.3	90.32	8–16	0.994	0.05 *	0.006	0.002
			172.9	109	30								
Tebuconazole	4	23.933	125	89	15	98.4	93.2	87.51	8–10	0.993	0.1	0.01	0.003
			250	125	20								
Epoxiconazole	4	24.521	192	138.1	10	99.18	100.88	87.43	12–16	0.998	0.6	0.01	0.003
			192	111	25								
Bromuconazole	5	24.939	173	145	15	85.00	87.84	89.24	13–15	0.985	0.2	0.012	0.004
			173	109	30								
Metconazole	5	25.569	125	89	20	91.5	92.5	90.92	6–8	0.996	0.15	0.012	0.004
			125	99	20								
Fenbuconazole	6	28.694	128.9	102.1	15	91.8	88.54	92.33	6–11	0.999	0.1	0.01	0.003
			197.9	129	5								
Difenoconazole	6	31.769	322.8	264.8	15	94.5	93.57	93.74	12–16	0.998	0.1	0.01	0.003
			264.9	202	20								

(*) Indicates the lower limit of analytical determination as mentioned by the EU, Rt: retention time, C.E: collision cell energy (volt), RSD Ave: relative standard deviation average, R^2^: correlation coefficient of regression line, MRL: maximum residue level (reported by European commission), LOQ: limit of quantitation, LOD: limit of detection.

**Table 2:** Determination of triazole derivative residue (ppm) in different imported wheat type grains from different countries of origin by GC-MS/MS.

Fungicide	Total Detected Sample	USA(S)		Canada(H)		Germany(H)		Germany(S)		Australia(H)		French(S)		Lithuania(H)		Polish(H)		India(S)		Brasilia(H)	
		Samples Detected *	Avg	Samples Detected	Avg	Samples Detected	Avg	Samples Detected	Avg	Samples Detected	Avg	Samples Detected	Avg	Samples Detected	Avg	Samples Detected	Avg	Samples Detected	Avg	Samples Detected	Avg
			%		%		%		%		%		%		%		%		%		%
Simeconazole	4	ND	---	ND	---	0.022 0.037	0.03	0.024	0.024	ND	---	0.014	0.014	ND	---	ND	---	ND	---	ND	---
							33.33		50.00				16.66								
Tetraconazole	---	ND	---	ND	---	ND	---	ND	---	ND	---	ND	---	ND	---	ND	---	ND	---	ND	---
Penconazole	27	0.015	0.015	0.077 0.047 0.023 0.044	0.047	0.034 0.053 0.047 0.052	0.046	0.051	0.051	0.032	0.032	0.061 0.042	0.051	0.043 0.030 0.041 0.040	0.038	0.087 0.051 0.016 0.017 0.023 0.021 0.022	0.033	0.041	0.051	0.063 0.043	0.053
			33.33		40.00		66.66		50.00		33.33		33.33		100.00		53.84		33.33		50.00
Hexaconazole	24	0.015	0.015	0.027 0.019 0.023 0.013 0.024	0.021	0.034 0.023 0.027 0.030	0.028	ND	---	ND	---	0.030 0.040 0.012	0.027	0.023 0.033 0.040 0.022	0.029	0.017 0.032 0.016 0.041 0.023	0.025	0.028	0.028	0.018	0.018
			33.33		50.00		66.66						50.00		100.00		38.46		33.33		25.00
Azaconazole	---	ND	---	ND	---	ND	---	ND	---	ND	---	ND		ND	---	ND	---	ND	---	ND	---
Cyproconazole	10	ND	---	0.042	0.042	0.077 0.154	0.115	0.046	0.046	ND	---	0.018 0.027 0.017	0.020	ND	---	0.027	0.027	0.025 0.042	0.033	ND	---
					10.00		33.33		50.00				33.33				7.69		66.66		
Diniconazole	11	0.012	0.012	0..022 0.014 0.018	0.018	0.026 0.014	0.020	0.027	0.027	ND	---	0.022 0.017	0.019	ND	---	0.024	0.024	ND	---	0.026	0.026
			33.33		30.00		33.33		50.00				33.33				7.69				25.00
Etaconazole	---	ND	---	ND	---	ND	---	ND	---	ND	---	ND	---	ND	---	ND	---	ND	---	ND	---
Propiconazole	---	ND	---	ND	---	ND	---	ND	---	ND	---	ND	---	ND	---	ND	---	ND	---	ND	---
Tebuconazole	24	0.027 0.043 0.012	0.027	0.020 0.021 0.019	0.020	0.062 0.067 0.077 0.082	0.072	0.098	0.098	0.063	0.063	0.048 0.028 0.039	0.038	0.052 0.054	0.053	0.034 0.055 0.021 0.026 0.062 0.016	0.035	ND	---	0.096	0.096
			100.0		30.00		66.66		50.00		33.33		50.00		50.00		46.15				25.00
Epoxiconazole	---	ND	---	ND	---	ND	---	ND	---	ND	---	ND	---	ND	---	ND	---	ND	---	ND	---
Bromuconazole	---	ND	---	ND	---	ND	---	ND	---	ND	---	ND	---	ND	---	ND	---	ND	---	ND	---
Metconazole	4	ND	---	ND	---	ND	---	ND	---	ND	---	ND	---	ND	---	0.015 0.013 0.012 0.011	0.012	ND	---	ND	---
																	30.76				
Fenbuconazole	7	ND	---	0.017	0.017	0.041 0.047	0.044	0.021	0.021	ND	---	ND	---	ND	---	ND	---	ND	---	0.013 0.016 0.026	0.018
					10.00		33.33		50.00												75.00
Difenoconazole	4	0.012 0.012	0.012	ND	---	ND	---	ND	---	ND	---	ND	---	ND	---	ND	---	0.022 0.024	0.023	ND	---
			66.66																66.66		

* Sample detected: the number of contaminated samples detected for each wheat group and fungicide; Total detected sample: the number of total contaminated samples for all wheat origins; Avg.: average residue of the detected samples in ppm; %: detected contaminated sample percentage to no. of samples analyzed for each wheat origin; ND: not detected.

**Table 3:** Determination of deoxynivalenol (DON) and T-2 toxins (ppb) in different imported wheat grains from different origin countries.

Mycotoxin	Total Detected Sample	Sample Serial Code																			
		USA(S)		Canada(H)		Germany(H)		Germany(S)		Australia(H)		French(S)		Lithuania(H)		Polish(H)		India(S)		Brasilia(H)	
		Samples Detected	x`	Samples Detected	x`	Samples Detected	x`	Samples Detected	x`	Samples Detected	x`	Samples Detected	x`	Samples Detected	x`	Samples Detected	x`	Samples Detected	x`	Samples Detected	x`
DON (ppb)	53	943.0 130.0 1008.0	693.7	1598.0 410.0 1123.0 840.0 912.0 639.0 626.0 1543.0 485.0 530.0	870.6	122.0 132.0 133.0 320.0 145.0 468.0	220.0	287.0 133.0	210.0	82.0 60.0 49.0	63.7	569.0 14.0 17.0 38.0 852.0 822.0	385.3	1806.0 1187.0 814.0 268.0	1018.8	388.0 501.0 825.0 661.0 314.0 460.0 658.0 347.0 230.0 250.0 379.0 71.0 212.0	407.4	24.0 34.0 64.0	40.7	1739.0 ND 36.0 34.0	603.0
T-2 (ppb)	54	139.8 131.2 120.1	130.4	117.5 124.0 138.2 128.4 127.1 140.4 135.6 131.6 109.7 122.9	127.5	112.4 129.1 121.4 107.4 131.1 130.6	122.0	118.5 123.0	120.8	123.5 122.3 112.9	119.6	138.2 145.5 140.5 125.0 123.6 121.7	132.4	1107.0 136.8 136.1 129.5	377.4	105.1 104.0 104.4 100.2 116.6 98.1 110.0 124.3 121.4 129.1 113.7 131.1 140.5	115.3	126.6 112.3 139.7	126.2	139.3 119 133.9 108.3	125.1

**Table 4:** Random representative sample origin, wheat type, code, and number of collected samples.

Nr.	Origin Country	Wheat Type	Code *	No. of Samples
1	American	Soft	USA(S)	3
2	Canadian	Hard	Canada(H)	10
3	German	Hard	Germany(H)	6
4	German	Soft	Germany(S)	2
5	Australian	Hard	Australia(H)	3
6	French	Soft	French(S)	6
7	Lithuanian	Hard	Lithuania(H)	4
8	Polish	Hard	Polish(H)	13
9	Indian	Soft	India(S)	3
10	Brazilian	Hard	Brasilia(H)	4
	Total	-	-	54

* Sample origins have been given a code to facilitate data analysis and the source of the samples was the Saudi Grains Organization (SAGO).

## Data Availability

Wheat (*Triticum aestivum)* were used in this study. It was withdrawn from the imported wheat in favor Saudi grains Organization.

## Sample Availability

Samples of the compounds are not available from the authors.
